# A method for encoding clinical datasets with SNOMED CT

**DOI:** 10.1186/1472-6947-10-53

**Published:** 2010-09-17

**Authors:** Dennis H Lee, Francis Y Lau, Hue Quan

**Affiliations:** 1 School of Health Information Science, University of Victoria, Human & Social Development Building A202, 3800 Finnerty Road (Ring Road), Victoria, BC V8P 5C2, Canada; 2 Edmonton Palliative/End of Life Program, Alberta Health Services, Seniors Health, Grey Nuns Community Hospital, 335 St. Marguerite Health Services Centre, 1090 Youville Drive, Edmonton, AB T6L 5X8, Canada

## Abstract

**Background:**

Over the past decade there has been a growing body of literature on how the Systematised Nomenclature of Medicine Clinical Terms (SNOMED CT) can be implemented and used in different clinical settings. Yet, for those charged with incorporating SNOMED CT into their organisation's clinical applications and vocabulary systems, there are few detailed encoding instructions and examples available to show how this can be done and the issues involved. This paper describes a heuristic method that can be used to encode clinical terms in SNOMED CT and an illustration of how it was applied to encode an existing palliative care dataset.

**Methods:**

The encoding process involves: identifying input data items; cleaning the data items; encoding the cleaned data items; and exporting the encoded terms as output term sets. Four outputs are produced: the SNOMED CT reference set; interface terminology set; SNOMED CT extension set and unencodeable term set.

**Results:**

The original palliative care database contained 211 data elements, 145 coded values and 37,248 free text values. We were able to encode ~84% of the terms, another ~8% require further encoding and verification while terms that had a frequency of fewer than five were not encoded (~7%).

**Conclusions:**

From the pilot, it would seem our SNOMED CT encoding method has the potential to become a general purpose terminology encoding approach that can be used in different clinical systems.

## Background

In October 2005, Canada Health Infoway recommended the Systematised Nomenclature of Medicine Clinical Terms (SNOMED CT) as the preferred reference terminology for recording patient data as part of the interoperable Electronic Health Record (iEHR) initiative. SNOMED CT is a comprehensive reference terminology that allows healthcare providers to record clinical encounters accurately and unambiguously. Multiple SNOMED CT concepts can be joined together to create post-coordinated expressions that allow users to record complex clinical conditions. Concepts are organised into 19 hierarchies such as body structures, clinical findings, events and procedures. The July 2008 release version of SNOMED CT contained over 388,000 concepts, 1.14 million descriptions and 1.38 million relationships, with regular updates every six months by the National Release Centers of the respective charter member countries [1]. Over the past decade there has been a growing body of literature on how SNOMED CT can be implemented and used in different clinical settings [2-6]. Yet, for those charged with incorporating SNOMED CT into their organisation's clinical applications and vocabulary systems, there are few detailed encoding instructions and examples available to show how this can be done and the issues involved.

This paper describes a heuristic method that can be used to encode clinical terms in SNOMED CT and an illustration of how it was applied to encode an existing palliative care dataset. The encoding method was first developed as part of a master's project and has since been expanded through several small-scale studies with different clinical datasets [7] and other unpublished analysis. This method has been further refined through a one-year pilot project to encode clinical terms from an existing palliative care information system in a Canadian healthcare organization into SNOMED CT.

### Project Background

The purpose of the project, "A Standards-based Palliative Care Information System (PCIS) for Alberta Health Services, Edmonton Zone," was to explore the adoption, use and impact of SNOMED CT. The objectives included creating a SNOMED CT palliative care subset, enhancing the PCIS with SNOMED CT and to determine the impact on quality of care, including clinician satisfaction and change management processes. As we did not have a predefined list of terms to encode with SNOMED CT, part of the process of developing the palliative care subset was to explore what data items in the PCIS could be encoded with SNOMED CT. As details of the pilot project are being published elsewhere, this paper only focuses on describing the encoding method that was used to derive the palliative care subset. This project had ethics approval from both the Edmonton Capital Health Region - Health Research Ethics Board (Health Panel, protocol Pro00005461) and University of Victoria Human Research Ethics Board (protocol 09-182).

**Figure 1:**
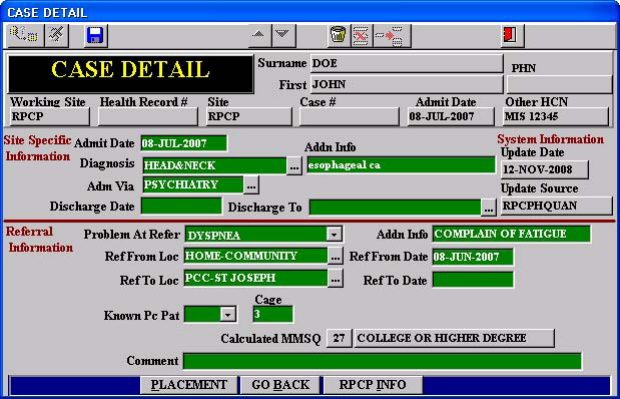
**Screenshot of the "Case Detail" screen of the Palliative Care Information System**.

The database schemas, allowable codes and anonymised free-text entries were extracted from the PCIS. The PCIS has two data fields, "diagnosis" and "problem at referral" that are used to encode the patients' clinical findings. There are 20 pre-defined diagnosis and 14 pre-defined problems at referral that could be selected from a pick-list. Sixteen of the diagnoses refer to cancer categories (e.g., "bone and connective tissue," "melanoma" and "eye, brain and other parts of the central nervous system") while four refer to non-cancer categories (i.e., neuromuscular, cardiopulmonary, infectious diseases and other). The pre-defined problem at referral includes findings such as "pain," "delirium," "nausea/vomiting" and "asthenia." Any additional details were recorded in free text fields labelled as additional information. Since there were only 34 pre-defined pick list items, the vast majority of this information was recorded as free text. A sample screenshot of the PCIS is shown in Figure 1. For this pilot, the July 31, 2008 International Release version of SNOMED CT was used.

## Method

**Figure 2:**
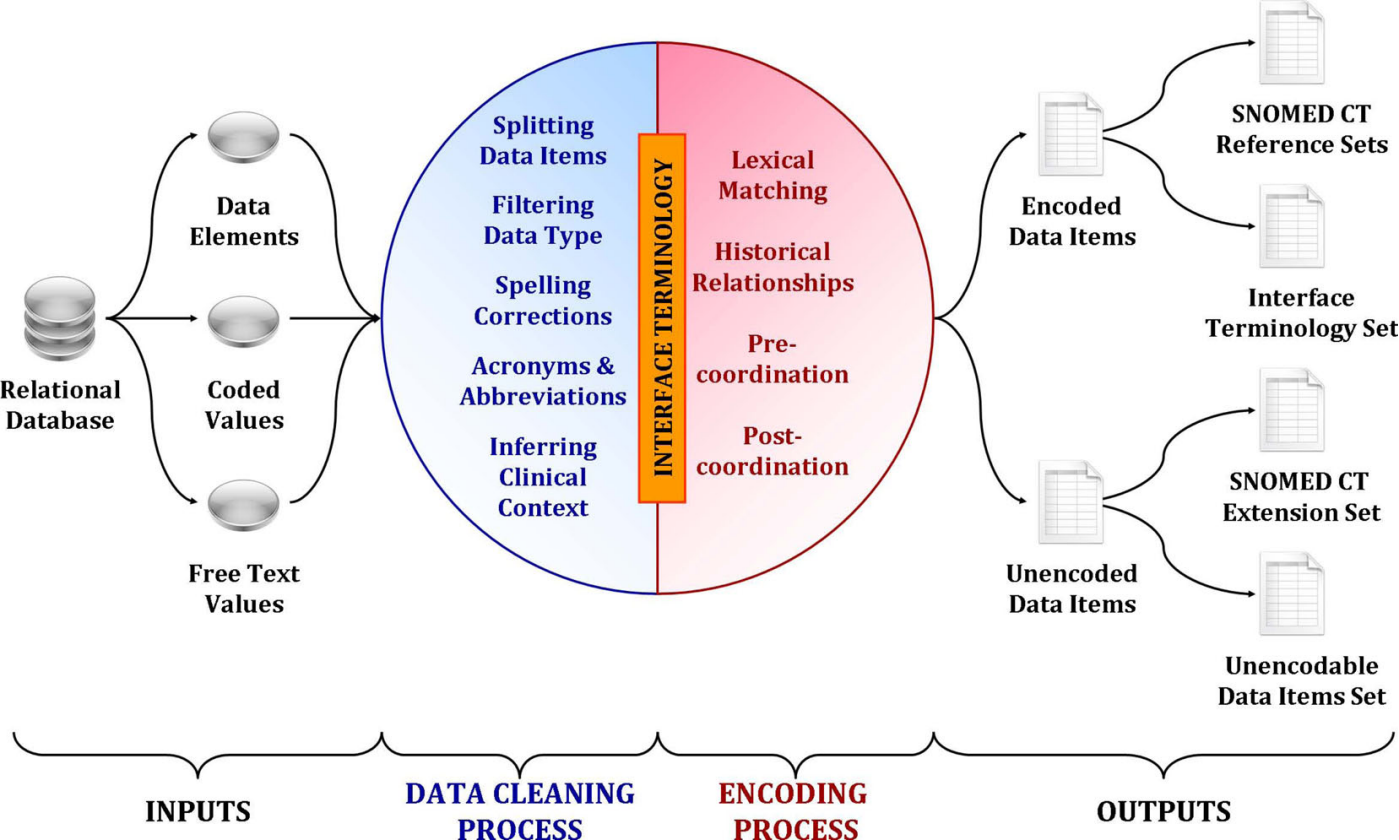
**An overview of the SNOMED CT encoding method**.

Our heuristic SNOMED CT encoding method has four parts: (a) identifying input data items; (b) cleaning the data items; (c) encoding the cleaned data items; and (d) exporting the encoded terms as SNOMED CT term sets. An overview of this method is shown in Figure 2. Three software tools are used in the encoding process - a batch matching algorithm, CliniClue Browser and Microsoft Excel. The batch matching algorithm reduces the amount of manual work by automating the matching of cleaned data items with SNOMED CT, the CliniClue Browser is used to manually look up concepts, while Microsoft Excel is used to view the results.

### Identifying Input Data Items

#### Identifying the Potential Data Items to be Encoded

The first step is to identify the potential data items in the database source for encoding. There are three types: data elements, coded values and free text values. Data elements refer to the name of input fields or can be thought of as a question (e.g., "Diagnosis," as in "What is the diagnosis of the patient?"). The answer can take the form of coded values or free text values. Coded values are answers that have been pre-defined and may be selected from a pick-list (e.g., "Lung cancer," or "Breast cancer"). If a patient has a diagnosis that has not been predefined, additional diagnosis details may be recorded in a free text field.

#### Preparing the List of Data Items

When extracting data items, it is important to keep an audit trail the data items to enable us to trace where the terms originated from. This audit trail should include the table name, data element name, data type as well as code and description if applicable. Screenshots of the application where the data item is used would also be useful in understanding the context in which it is used.

### Extracting Data Elements

The names of data elements can be extracted manually by viewing the database schema and copying each data element name, or using a database management software application to export the schema into a text file or spreadsheet. Data elements in a database can be classified as those relating to the clinical encounters, identifiers or audit trail. The clinical encounters refer to data elements such as patient name, diagnosis and gender. The identifiers refer to data elements that are usually the primary or foreign keys of tables. The audit trail refers to data elements such as user account info, audit logs on whom added or edited a record and when it was changed. The audit trail can also refer to data elements such as the status of a coded value whether it is active or not. It is unlikely that identifiers and audit trail data elements can be encoded since SNOMED CT was not designed for this purpose.

### Extracting Coded Values

Coded values are generally stored in lookup tables. These lookups are usually external tables in the database and are separate from the application although there are instances in which the coded values are embedded within the software itself. If the coded values are embedded within the program code, it may be necessary to manually copy each coded value if the software does not include an export feature. For lookup tables that are recorded in the database, depending on its design, the coded values may be recorded in one table or the coded values for each data element may be in individual tables. When extracting coded values, it is important to distinguish between the code (i.e. value) and code description (i.e. value meaning). For example, "Male" is a code description and may be represented with the alphabetic code "M" or a numeric code such as 1. When encoding terms in SNOMED CT, the code description should be used but it is important to be able to link the code description back to the code. Encoding coded values with SNOMED CT can be considered a form of mapping.

### Extracting Free Text Values

The first step in extracting free text values is to identify the source table and data element name. Not every data element with free text should be extracted. For example, free text values from data elements such as names and addresses should not be extracted because they cannot be encoded in SNOMED CT. Users should browse through the records to determine what data are contained in a data element as it may not be obvious by looking at just the data element name. Once the data element has been identified, the unique terms should be extracted and their frequencies tabulated. Having this information is important because more effort should be placed on frequently occurring terms.

### Collating Data Items

Once the data items have been identified and extracted from various data sources, they should be collated and sorted by frequency. As data cleaning and encoding are very time-consuming processes, the terms should be grouped in batches by frequency so more time can be spent on frequently occurring terms. It is not necessary to clean every single term before proceeding to the encoding step as data cleaning can be a time-consuming process. Shortcuts and lessons learned from an earlier batch should be applied to the next round to improve the process.

### Cleaning the Data Items

**Figure 3:**
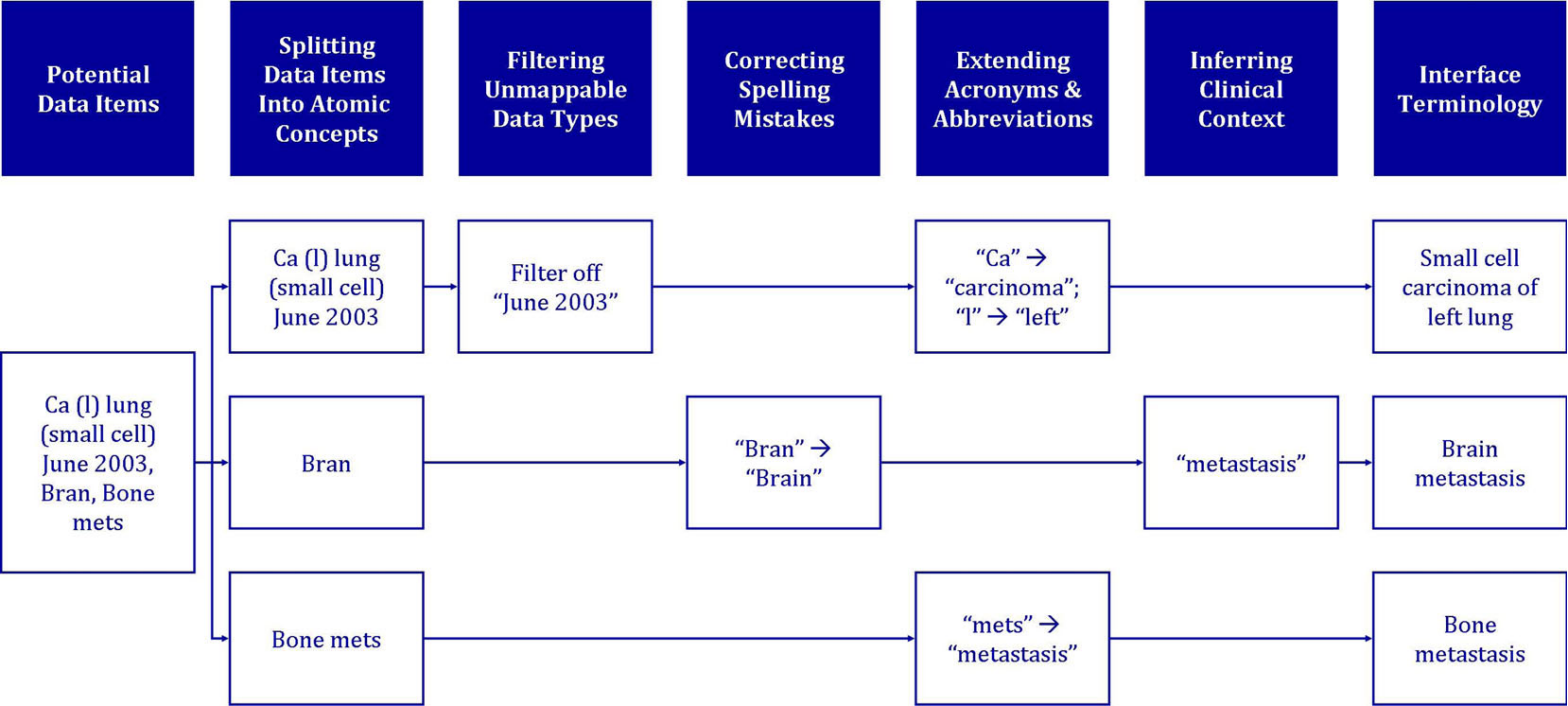
**An overview of the data cleaning process with examples**.

The data cleaning process ensures the data items are consistent and accurate. Once these terms are cleaned, they essentially represent an interface terminology, which helps "support interactions between healthcare providers and computer-based applications."[8] The three types of data items require different extents of data cleaning. Coded values require minimal cleaning as they have already been vetted by the organisation before they are added as pick-list items. On the other hand, free text values require the most cleaning as there is often no restriction on what can be recorded. Data elements that need to be encoded also require some cleaning as data element names may use abbreviations or acronyms, or if multiple words are used, spaces are removed or underscores are used to separate words. While cleaning the data items, it is important to maintain an audit trail so as to be able to link them back to the original term. See Figure 3 for an overview of the cleaning process.

#### Splitting the Data Items

There may be instances where coded values or free text values contain multiple clinical conditions. While it may be possible to encode these clinical conditions as a single complex SNOMED CT expression, it is advisable to break these descriptions into atomic concepts as they will be easier to encode and will be more useful in queries. The delimiters used to separate multiple clinical conditions can vary depending on the database. Common delimiters are semicolons (;), commas (,) and full stops (.). In addition, words such as "and," "with" and the symbol ampersand (&) may be used as delimiters to join multiple concepts. The use of delimiters to split terms can be done automatically although it should be manually reviewed as there might not be a standardised delimiter and terms may be split incorrectly. For example, a full stop may not signal the end of a sentence but refer to an abbreviation (e.g., "abd." as in "abdomen"). If a coded or free text value contains a clinical condition that is mixed with other types of data (e.g., numbers, dates), it is necessary to split them up. For example, with the phrase "lung cancer onset in June 2003," it is necessary to split up the actual diagnosis (i.e., "lung cancer") from the date of the diagnosis (i.e., "June 2003"). Data elements and coded values generally do not require any splitting of terms because they usually represent atomic concepts. On the other hand, free text values frequently include multiple terms. It should be noted that the splitting of data items can be tricky as SNOMED CT does include some concepts that refer to multiple clinical conditions such as "26298008|Diabetic coma with ketoacidosis (disorder)|". The splitting of such terms can cause a loss in semantics.

#### Filtering Data Types

SNOMED CT is designed to encode clinical encounters but not other types of data such as names, dates, numbers and measurements. Although an exact date in a patient record cannot be encoded with SNOMED CT, it is possible to encode the term as a past medical history. While it was not possible to encode the exact date such as of "January 1, 1880," it is possible to encode the date as a past medical history by setting the "408731000|Temporal context (attribute)|" as "410513005|In the past (qualifier value)|". The decision on whether to encode dates as a past medical history will have to be made individually by each organisation.

As such, these data types should be filtered and not be part of the potential list of terms to be encoded. The data that is excluded should be kept as part of the provenance information. Although the excluded data types cannot be encoded in SNOMED CT, it may be possible to encode them using an information model, such as HL7's Reference Information Model. The splitting of terms and filtering of data types may require several rounds to filter out all unsuitable terms.

#### Spelling Corrections

One of the major hindrances to lexical matching is spelling mistakes. All three types of data items should undergo a spelling correction algorithm. An index of words found in the data elements, coded values and free text values can be generated and compared to the index of words found in SNOMED CT. A simple comparison of these indexes can quickly narrow down the potentially problematic words. Data elements often do not contain spaces in their names or underscores are used to separate words. For example, the data element "First Name" may be labelled "FName," "FirstName" or "First_Name." These forms will decrease the likelihood of finding a lexical match.

#### Acronyms and Abbreviations

The inconsistent use of abbreviations and acronyms also diminishes successful lexical matching. For example, "ca" can mean "cancer," "carcinoma" or even "calcium." Organisations may use abbreviations and acronyms that are only understood locally. It is necessary to spell out all abbreviations and acronyms to remove any ambiguity as well as to improve the chances of finding lexical matches.

#### Inferring Clinical Context

There are occasions where it is necessary to infer the underlying clinical condition for a given term. This ambiguity may be the result of the process of splitting terms into atomic concepts, the loss of context, or that the original term was in itself ambiguous. For example, in Figure 3, after the previous data cleaning steps, we ended up with three main concepts: "small cell carcinoma of left lung," "brain" and "bone metastasis." It is necessary to infer that "brain" refers to "brain metastasis" and not just the region of the brain. In order to find an appropriate concept in SNOMED CT, it is difficult to deal with terms such as "brain metastasis," "metastatic brain cancer" and "metastatic brain disease." The question to be asked here is: What does it mean? Is it a primary carcinoma or neoplasm of the brain or is it neoplasm metastases to the brain? SNOMED CT includes different concepts to describe each situation so it is important that the intent of the term used is understood. This can be inferred by looking at the context, comparing the clinical statement with other similar statements, or checking with the clinician who made the remarks. Another example of the loss of context is when a data element is "Cancer region," that is to ask, "Where is the location of the primary neoplasm?" The answer may be "brain" or "bone," but when preparing a data item for encoding, it should reflect the full context, as in "brain cancer" or "bone cancer." The difference between a body structure and clinical condition will make a difference in the selection of a concept.

#### Initial Interface Terminology

The cleaned terms are then referred to as an "initial" interface terminology since they are made up of the cleaned original terms from the local database that are to be encoded in SNOMED CT. After the encoding process, we will derive a "final" interface terminology which contains all of the preferred terms used by clinicians in data entry. These preferred terms may include the local cleaned terms (provided that the cleaning process ensures that the terms can be made consistent or standardised beyond the local organization) or their encoded SNOMED CT preferred/synonym terms depending on clinician preference. If the intent is to replace all local terms with SNOMED CT concepts in the clinical system, then this interface terminology can serve as a historical index to facilitate the transition.

##### 1.1 Encoding the Cleaned Data Items

**Figure 4:**
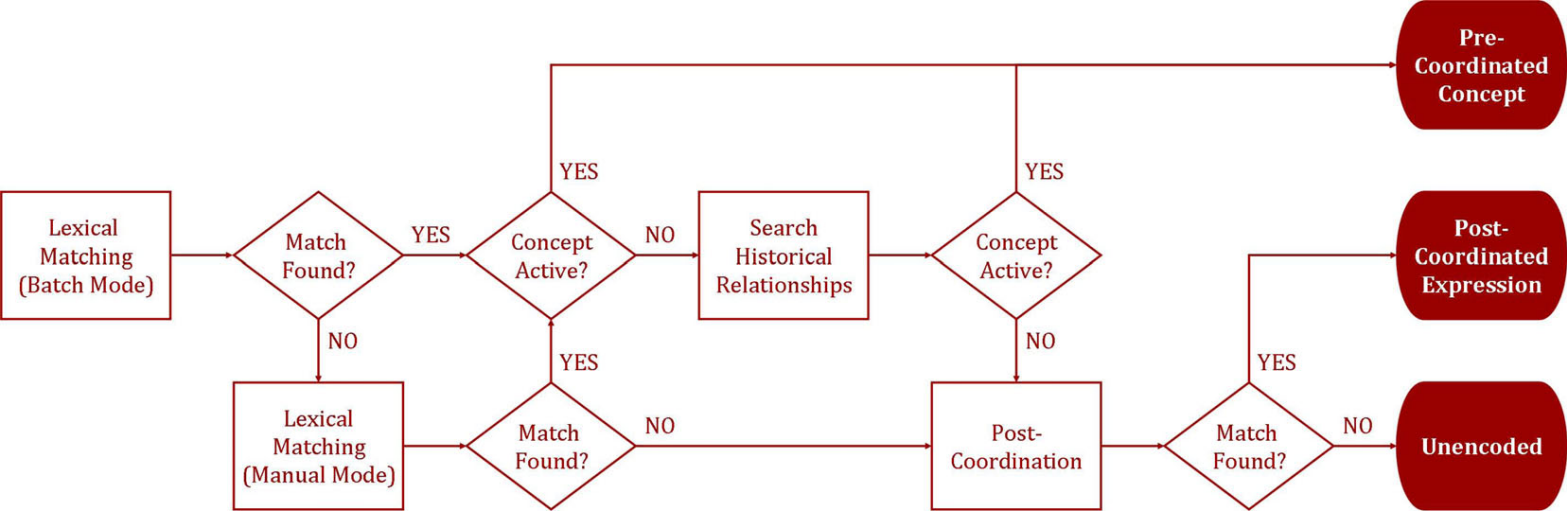
**An encoding process flowchart**.

The next step is the encoding process. Figure 4 shows this process in a flowchart. The first step is to locate a lexical match through a batch mode. If a match is found and the concept is active, the term can be encoded with a pre-coordinated concept. If the concept is inactive, an attempt will be made to locate an active concept (i.e., concept status "current") through the historical relationships. If no match is found using the batch mode, a manual search will be done. If the term cannot be matched using a pre-coordinated concept, an attempt will be made to represent the term with multiple concepts, or post-coordination. If no post-coordinated expression can adequately represent the term, it is considered unencodeable.

#### Lexical Matching of Terms

Lexical string matching is our method of locating the SNOMED CT concepts. There are two steps involved. First is a batch mode where data items are automatically matched using a batch matching algorithm. Second is a manual mode where all outstanding unmatched terms are manually matched using the CliniClue Browser.

### Batch Mode

The batch matching algorithm is a set of structured query language (SQL) queries that are used to search for SNOMED CT terms. The batch matching algorithm takes a list of data items, normalises these terms, and matches both the original data items and normalised terms against the original SNOMED CT descriptions as well as a normalised set of SNOMED CT descriptions. The normalisation process [7] involves removing punctuation, prefixes, stop words, exclude words as well as stemming (searching for the base (root) form of inflected word) through the UMLS SPECIALIST Lexicon inflections table [9]. Punctuation, stop words and exclude words are excluded as they can decrease the success of finding lexical matches. The list of stop words and excluded words used are recommended by SNOMED CT [10]. The removal of these words helps improve the results as well as the time it takes to search for matches. The batch matching algorithm can return results by exact match, match all words, or partial match. Exact matches are the gold standard and occur when all words are found in the SNOMED CT description and are in the same order as the data item. Match all words generally produces good results and occurs when all the words in the data item are found in the SNOMED CT description although not necessarily in the same sequence as the data item. The SNOMED CT description may contain additional words. However, if the data items are short or the words are common, there can be many potential matches. Partial matches are the least accurate as only one word needs to be present.

The purpose of using a batch matching algorithm is to streamline the process of locating potential SNOMED CT concepts as manually looking up each term is a time-consuming process. All results of the batch matching algorithm should be manually reviewed to ensure that appropriate concepts are selected.

### Manual Mode

Terms that cannot be encoded using the batch mode are searched for manually using the CliniClue Browser. When a manual search is conducted, synonyms and other phrases are often used in an attempt to find relevant SNOMED CT concepts. For example, SNOMED CT does not include a concept for "non-melanoma of the skin." Other synonyms or more general terms such as "skin disorder," "lentigo," "acquired melanocytic nevus" may be used instead. If a pre-coordinated concept cannot be found, post-coordination will be attempted.

### General Guidelines for Selecting Concepts from Hierarchies

We developed some basic guidelines on which concepts to select if the should be multiple exact matches. This is most often the case with concepts from the "404684003|Clinical finding (finding)|" hierarchy and subtypes of "118956008|Body structure, altered from its original anatomical structure (morphologic abnormality)|". For example, the term "fracture" can refer to the descriptions "125605004|Fracture|" or "72704001|Fracture|". Taking a look at the fully-specified name, the former refers to "125605004|Fracture of bone (disorder)|" while the latter refers to "72704001|Fracture (morphologic abnormality)|". The clinical findings should generally be preferred over the morphologic abnormality concept. A closer look shows that "72704001|Fracture (morphologic abnormality)|" is a defining attribute of "125605004|Fracture of bone (disorder)|" and is linked using the concept model attribute "116676008|Associated morphology (attribute)|". If there is only a morphologic abnormality concept, it should be post-coordinated with a focus concept of "64572001|Disease (disorder)|" and linked with the concept model attribute "116676008|Associated morphology (attribute)|". Another example is the term "morphine," which in SNOMED CT can refer to either "73572009|Morphine (product)|" or "373529000|Morphine (substance)|". The defining attributes shows that "73572009|Morphine (product)|" is linked to "373529000|Morphine (substance)|" through the concept model attribute "127489000|Has active ingredient (attribute)|". If the context of morphine refers as medications or opioids, then the product concept should be used.

#### Historical Relationships

The historical relationships in SNOMED CT are used to relate inactive concepts to active concepts. There are six historical relationships: "149016008|MAY BE A (attribute)|", "384598002|MOVED FROM (attribute)|", "370125004|MOVED TO (attribute)|", "370124000|REPLACED BY (attribute)|", "168666000|SAME AS (attribute)|" and "159083000|WAS A (attribute)|". In lexical matching, all concepts are searched regardless of concept status. When an inactive concept is retrieved, the historical relationships may point to an active concept. If a concept retrieved is inactive, the historical relationships are used to locate an active concept. The results of the batch matching algorithm need to be manually verified to ensure the appropriate concepts are selected.

#### Pre-coordinated Terms

There are instances whereby a SNOMED CT concept represents multiple findings. An example is "nausea and vomiting." While the term could be split into two separate atomic terms and be encoded separately as "422587007|Nausea (finding)|" and "422400008|Vomiting (disorder)|", there is a single concept in SNOMED CT "16932000|Nausea and vomiting (disorder)|". Whenever a pre-coordinated concept is available, that should be the first choice. If the pre-coordinated concept is a primitive concept, an alternative is to create a post-coordinated expression to ensure the necessary semantics are recorded. The reason is that terms encoded with primitive concepts are more difficult to query and test for equivalency.

#### Post-coordinated Terms

There are instances when pre-coordinated concepts do not adequately convey the meaning of a data item. It is then necessary to post-coordinate multiple concepts. There are three types of post-coordination: combination, qualification and refinement. Combination refers to combining two concepts. Qualification refers to appending a qualifying characteristic to a concept. Refinement is similar to qualification but instead of appending a qualifying characteristic to a concept, a subtype of a defining characteristic associated with a concept is selected. When creating post-coordinated expressions, it is important to adhere to the guidelines in the SNOMED CT concept model [11]. There are, however, occasions where it may be necessary to use unapproved or unsanctioned attributes to qualify concepts with qualifier values that would otherwise be unusable. There are areas in SNOMED CT that have not been fully defined and require further exploration on how to deal with the encoding of certain types of clinical findings. Caution must be exercised when using unapproved attributes since their status can change in new releases which may require modifications to the expressions. It is therefore important to submit new attributes to the IHTSDO Editorial Board - Content Committee to ensure these extensions are recognised internationally. It should be noted that only seven top-level hierarchies are eligible for post-coordination at present. Therefore not every SNOMED CT concept can be post-coordinated through the concept model.

#### Unencodeable Terms

If no pre-coordinated concept or post-coordinated expression can adequately represent a data item, and if there is no desire to create no extension, that term is marked as unencodeable.

### Exporting the Encoded Terms as SNOMED CT Term Sets

**Figure 5:**
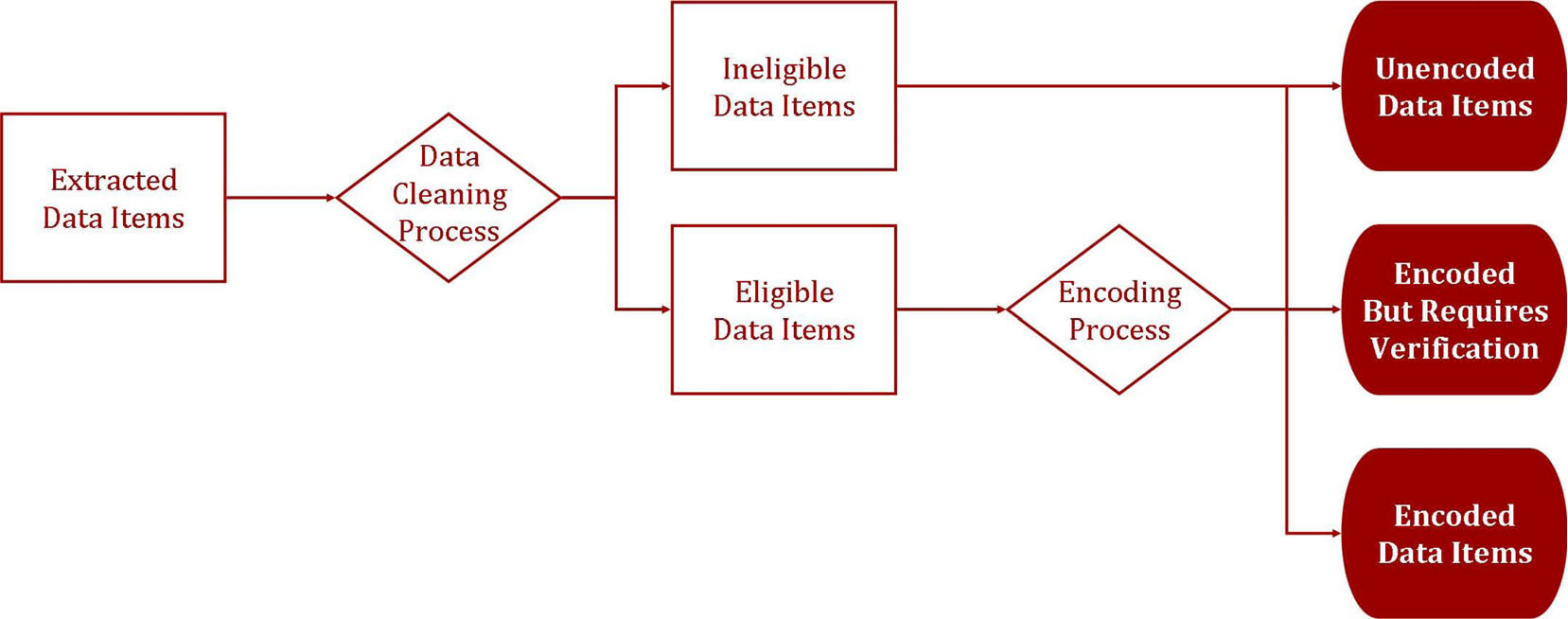
**The types of encoding outputs from the data cleaning process**.

Once all the terms have undergone the encoding process, they can be categorised as encoded and unencoded terms. The encoded terms are then used to generate a SNOMED CT reference set and the "final" interface terminology. The unencoded terms that occur frequently but have no SNOMED CT equivalent can be submitted for inclusion into a future release of SNOMED CT through the SNOMED CT extension set. Other unencoded terms that occur rarely may be classified as an unencodeable term set if they are not to be submitted as part of the extension set. A summary of the types of encoding outputs from the data cleaning process is shown in Figure 5.

#### Encoded Terms

### SNOMED CT Reference Set

The reference set mechanism in SNOMED CT is one method for filtering and arranging concepts for specific domains or use cases. By bundling concepts from a domain or use case as a reference set, these concepts are portable and may be distributed to other organisations that have similar needs [10].

### Interface Terminology Set

The interface terminology set refers to the encoded local terms and their corresponding SNOMED CT terms and expressions. The interface terminology set may be distributed in a number of formats and structures depending on the purpose and needs. For example, it can be distributed as a single table with the interface terminology in one column and the target SNOMED CT expression in another column. The SNOMED CT release version, long normal forms and short normal forms should also be recorded. The recording of the long and short normal forms can improve the performance of testing for equivalency and subsumption as well as check for changes between each SNOMED CT release.

#### Unencoded Terms

### SNOMED CT Extensions Set

The SNOMED CT extension mechanism allows organisations to develop their own SNOMED CT concepts "in accordance with the data structures and authoring guidelines applicable to SNOMED CT" [12] by requesting a seven-digit namespace identifier from the IHTSDO. The organisation should also submit the new concepts for possible inclusion into the core SNOMED CT releases.

### Unecodeable Term Set

There will inevitably be terms that are unencodeable. The reasons include: (a) ambiguous terms that cannot be clarified; (b) rare occurrence of terms that would not justify being added to an extension set; (c) data element names that refer to administrative purposes or audit logs. Also, depending on the scope of a study and its timeline, one may run out of time when there is a large amount of data to be encoded, especially free-texts that require cleaning. These terms should be bundled into an unencodeable term set so they can be re-examined later to derive a solution.

## Illustrative Example

The following section describes how the method was used to encode a palliative care dataset.

### Palliative Care Data Items as Inputs

The palliative care database had 33 tables. There were 211 data elements, 146 of which were clinical encounter related that were eligible for encoding in SNOMED CT. Another 26 and 39 elements were identifiers and audit trail, respectively, which were ineligible for encoding. We also extracted a total of 145 coded values from 15 data elements. These were mostly demographic information such as marital status and gender, clinical findings such as diagnosis and problems, and medication such as opioids and route of administration. Only two data elements, "Diagnosis" and "Problem-at-referral," contained free text values that were relevant. Other free text values such as the name of providers or care location were not relevant. For "Diagnosis," there were 24,356 (70.9%) patient records with free text values, while "Problem-at-referral" had 12,892 (37.5%) records with free text values. In all, we extracted 37,248 free text values from the anonymised database.

### Cleaning the Palliative Care Data Items

#### Splitting Terms

The 37,248 "Diagnosis" and "Problem-at-referral" terms were split into 58,829 atomic terms, of which 8,582 were unique. They were unique in the words present in the term and the order of the words but not necessarily in the concept they represented.

#### Filtering Data Types

The majority of free text values that were filtered from the "Diagnosis" and "Problem-at-referral" data elements referred to provider locations and were not encoded. There were also terms that included dates. For example, the date "feb 07" was filtered from "AML - feb 07."

#### Spelling Corrections

There were over 2,000 spelling mistakes with the free text. For example, the word "adenocarcinoma" was misspelled in over 30 ways in the free text diagnosis field. An index of words used in the free text was prepared and compared against the words found in SNOMED CT. This allowed us to quickly narrow down the potentially problematic words.

#### Acronyms and Abbreviations

Acronyms and abbreviations were common throughout the free text values and they were extended to the full long form. The previous example of filtering data types, "AML" contained an acronym, which stands for "acute myeloid leukemia." Examples of abbreviations included "abd" for "abdomen" and "mets" for metastasis.

#### Inferring Clinical Context

There were many terms that required clarification. An example was "appetite." Nearly 400 free text diagnoses included this term. In this instance "appetite" was ambiguous, unlike others such as "decreased appetite," "loss of appetite," "poor appetite" or "reduced appetite." The term "appetite" by itself was ambiguous so we asked the clinician who informed us that it meant the negative (i.e., "reduced" or "loss" of appetite).

#### Initial Interface Terminology

The initial set contained all of the cleaned data elements, coded values and free text values to be used as the data items in the encoding process. In the case of free text values for "Diagnosis" and "Problems-at-referral," there were different phrases found that referred to the same concepts such as diseases, findings, procedures and conditions.

### Encoding the Palliative Care Data Items

#### Lexical Matching of Terms

We ran the initial interface terminology set through the batch matching algorithm. We configured the algorithm to conduct only exact matches as the words in the initial term set were common and short. If a term contained just a few words and the words were common (words that occur frequently in SNOMED CT such as finding or disorder), there would be many results for match all and partial matches. For example, one of the terms that needed to be encoded was "lung cancer." In the "match all" algorithm, all the words in the source and target terms need to be present although not necessarily in the same order. A search for "lung cancer" would produce results such as "162573006|Suspected lung cancer (situation)|", "254632001|Small cell carcinoma of lung (disorder)|", "94391008|Secondary malignant neoplasm of lung (disorder)|" and "429011007|Family history of malignant neoplasm of lung (situation)|", all of which were not the appropriate matches. In this case study we did not limit the lexical matching to just concepts from the "404684003|Clinical finding (finding)|" hierarchy as the free text terms included references to events, procedures as well as family history. Limiting the search to a specific hierarchy may reduce the number of inappropriate matches, but it may also inadvertently leave out valid matches. This is especially true with free text, as a wide range of information such as procedures and events may be recorded in a field that has been designated as diagnosis.

#### Batch and Manual Matching of Terms

The batch lexical matching algorithm returned 37, 74 and 2,002 unique exact matches for the data elements, coded values and free text values respectively. The 2,002 unique free text matches represent 70.7% (41,603 of 58,829) of all free text values. Even though 2,113 terms were exactly matched, not all were appropriate and many still needed to be verified manually, which reduced the number of terms that were exactly matched correctly. The remaining unmatched terms were looked up manually. There were instances where the algorithm produced multiple exact matches for a single term. For example, "aspiration" was exactly matched to three concepts: "113049018|Aspiration|", "195646019|Aspiration|" and "415870011|Aspiration|". In this case, "aspiration" referred to "68052005|Pulmonary aspiration (finding)|", "14766002|Aspiration (procedure)|" and "278847003|Endotracheal aspiration (qualifier value)|". It was important to understand the context in which the term "aspiration" was used. Even though the "Diagnosis" field contained clinical findings, it had on occasions included procedures. Therefore we could not determine for sure whether the term referred to a clinical finding or procedure.

#### Finding Historical Relationships

The batch lexical matching algorithm produced 104 matches with SNOMED CT terms that were inactive, which required looking up their historical relationships to find the current concepts. An example was the data item "nocturia" where we found an exact match "221079019|Nocturia|". This description, however, belonged to an inactive concept (duplicate) "139394000|Nocturia (finding)|". By querying the relationships table, we found "139394000|Nocturia (finding)|" was the same as "6408001|Finding of nocturia (finding)|" and should be used instead.

#### Pre-coordinated Concepts

Of the 58,829 data items included in the encoding process, we were able to pre-coordinate 48,102 (81.77%) of them with 1,471 concepts. The majority of the pre-coordinated SNOMED CT concepts found were disorders, clinical findings and procedures matched to the free text values contained in the "Diagnosis" and "Problem-at-referral" field. The remaining pre-coordinated concepts covered different SNOMED CT hierarchies including observable entities, situations, persons, products and environments.

#### Post-coordination of Terms

For data items that could not be encoded with pre-coordinated concepts, attempts were made to encode them using post-coordination. Qualification and refinement were the most common forms of post-coordination used; combination was only used to establish two of the diagnosis categories for aggregation purposes. We manually encoded 1,373 (2.33%) data items with 468 SNOMED CT post-coordinated expressions.

### Qualification

In our encoding process, the concept model attributes that were used for qualification were "246112005|Severity (attribute)|", "263502005|Clinical course (attribute)|", "246456000|Episodicity (attribute)|", "408731000|Temporal context (attribute)|", and "408729009|Finding context (attribute)|". For example, to encode "severe asthenia" involves the concepts "13791008|Asthenia (finding)|" and "24484000|Severe (severity modifier) (qualifier value)|". These two concepts were linked with the concept model attribute "246112005|Severity (attribute)|".

### Refinement

In our encoding process, refinement was used to indicate a specific body structure through the "363698007|Finding site (attribute)|" and "116676008|Associated morphology (attribute)|" concept model attributes. For example, to encode "sarcoma of leg," the concepts "372151005|Sarcoma - category (morphologic abnormality)|" and "30021000|Lower leg structure (body structure)|" must be linked together with the concept model attribute "363698007|Finding site (attribute)|".

#### Unencoded Terms

There were different reasons some of the terms from the database were not encoded. First, we excluded 572 free text values that contained unsuitable data types which mostly consisted of such as health care provider locations and room numbers. No coded values were excluded as we selectively extracted only fields that were relevant. Second, we encoded 3,634 terms but they required further verification. Third, we encoded 1,002 terms but they required further post-coordination in order to fully represent the terms encoded. Fourth, five terms were ambiguous in their meaning thus required clarification from clinicians. Lastly, manual encoding was a tedious process that had consumed a great deal of time and effort. Only terms with a frequency of five or more were cleaned and encoded leaving 3,956 terms unencoded.

#### SNOMED CT Encoded Palliative Care Terms as Outputs

### Encoding Summary Statistics

In all, the original palliative care database contained 211 data elements, 145 coded values and 37,248 free text values. For data elements, 32 terms were encoded, 65 were ineligible and 124 remain unencoded. For coded values, all 145 were eligible for encoding. At present, 74 have been encoded while 71 remain unencoded. For free text values 37,248 terms were extracted and after splitting the terms into atomic concepts (58,829) and filtering data types (507), we were left with 58,272 terms. The initial batch matching algorithm encoded 41,603 of these terms and after verifying them and manually encoding the outstanding terms using both pre-coordination and post-coordination, a total of 49,475 (~84%) terms were encoded. All terms with a frequency of five and higher were successfully encoded. Another 4,891 (~8%) require further encoding and verification while 3,956 (~7%) remain unencoded. A summary of the types of encoding outputs and their respective frequencies is shown in Table 1. A summary of the extent of pre/post coordination done in encoding the free text values is shown in Table 2. Examples of unencoded terms are available in Table 3.

**Table 1:** A summary of the types of encoding outputs and their respective frequencies

Source		Extracted	Cleaned	Eligible	Ineligible	Encoded	Unencoded	Verification Required
Data Elements		211	211	146	65	32	124	0
Coded Values		145	145	145	0	74	71	0
Free Text Values	Unique	15,075	8,582	8,436	146	1,939	3,368	1,129
	Total	37,248	58,829	58,272	507	49,475	3,954	4,844

**Table 2:** A summary of pre/post coordination in encoding free text value

No	Encoding	Unique	Synonyms	Total	Percentage
1.	Pre-coordination	1,471	3,181	48,102	81.77%
2.	Post-coordination	468	691	1,373	2.33%
3.	Ambiguous	4	4	5	0.01%
4.	Requires post-coordination	693	693	1,002	1.70%
5.	Requires verification	342	342	3,634	6.18%
6.	Incorrect syntax or encoding	121	155	250	0.42%
7.	Unencoded	3,370	3,370	3,956	6.72%
8.	Unencodeable	146	146	507	0.86%
	**Totals**	**6,615**	**8,582**	**58,829**	**100.00%**

**Table 3:** Sample coded data values that could not be encoded with SNOMED CT

No	Term	Source	Comments
1.	Psychosocial distress	Problem at Referral	A possible SNOMED CT concept is "271596009|Mental distress (finding)|" but "mental" does not convey the same meaning as "psychosocial."
2.	Symptom management	Reason for Referral	The only concepts that contained the words "symptom" and "management" were "395087001|Pain and symptom management (procedure)|", "413742003|Cancer pain and symptom management (procedure)|", "410357008|Signs/symptoms-physical case management (procedure)|" and "410356004| Signs/symptoms-mental/emotional case management (procedure)|" but they were too specific.
3.	Education unknown	Highest Level of Education	Other values such as "college and university [education]" were encoded with "224300008|Received university education (finding)" but we could not find a term for "education unknown."
4.	Internal medicine	Admission Via	This data field is used to indicate the admission source. Other values such as "emergency" and "surgeon" were encoded with "305226003|Admission by Accident and Emergency doctor (procedure)|" and "305293001|Admission by surgeon (procedure)|" but we could not find a corresponding term for admission by internist.
5.	No medication	Medications	The closest term we could find was "371900001|Medication not administered (situation)|" but the meaning is slightly different.

### Final Interface Terminology

One of the outputs from the encoding process was the "final" interface terminology that was encoded with the appropriate SNOMED CT terms. The intent of this interface terminology was to allow clinicians to adopt a set of preferred terms that closely resemble what they often use, thus minimising the changes required when adopting SNOMED CT. It was clear from our encoding of the free text "Diagnosis" and "Problem-at-referral" values that often many variations of the same terms were recorded. As such, this provided an opportunity to harmonise these terms by selecting one standard term either using the SNOMED CT preferred/synonym or one of the cleaned local variants. Our encoding of the free text values also showed overlapping of the matched SNOMED CT terms between the two lists. Therefore we merged them into a common list to further sort and aggregate the SNOMED CT terms where feasible. See Table 4 for a sample list of the final interface terminology set with their corresponding SNOMED CT terms.

**Table 4:** Sample list of interface terminology with the corresponding SNOMED CT terms.

No	Local Terms	SNOMED CT Descriptions	SNOMED CT Concepts
1.	Dyspnea (951) SOB dyspnea (1) Short of breath (8) Shortness of breath (329) SOB (7)	Breathless Breathlessness Dyspnea Shortness of breath SOB - Shortness of breath	267036007|Dyspnea (finding)|
2.	Chronic obstructive airway disease (1) Chronic obstructive lung disease (1) Chronic obstructive pulmonary disease (1) COPD (200) Obstructive lung disease (11)	CAFL - Chronic airflow limitation CAO - Chronic airflow obstruction Chronic airflow limitation Chronic airflow obstruction Chronic airway disease Chronic airway obstruction Chronic obstructive airway disease Chronic obstructive lung disease COPD COPD-Chronic obstructive pulmonary disease	13645005|Chronic obstructive lung disease (disorder)|
3.	Caregiver burn out (3) Caregiver exhausted (3) Caregiver exhaustion (15) Caregiver stress (72) Caregiver stressed (3)	Care giver role strain	129891007|Caregiver role strain (finding)|

The numbers represent the frequencies of occurrence for the local terms in the source database.

## Results and Discussion

Despite growing interests from healthcare organisations to incorporate SNOMED CT as a reference terminology into their clinical information systems, there is still a lack of adequate guidance and examples on how one can and should implement this terminology standard. This is in spite of the effort from such groups as the IHTSDO to publish detailed SNOMED CT reference, technical, user and style guides to help with the process. Similarly, many of the educational workshops and tutorial materials in SNOMED CT available to date tend to focus on high level concepts and general approaches. While such training is a necessary first step for those just learning about SNOMED CT, it lacks the detail, tools and real-life examples for those who have to implement and support SNOMED CT in existing clinical systems.

As the majority of the effort was spent on data cleaning, one of the next steps would be to incorporate natural language processing algorithms to help automate more of the cleaning as well as the encoding. It is scalable to the point of available manual resources as all encodings need to be manually reviewed and post-coordination is currently completed manually.

The method described and applied in this paper takes a database of local clinical terms to identify the data items, clean and encode these terms, then export them as SNOMED CT encoded or unencodable term sets. Also produced are summary and detailed reports on the encoding results and statistics as outputs. To illustrate how it works, this method was applied in a pilot project to encode an existing palliative database, producing the corresponding term sets and report outputs. There are many terminology browser and encoding tools on the market [13], but many are proprietary without revealing the underlying methods and algorithms used. Therefore we believe our open approach can help advance the knowledge and experience in implementing SNOMED CT with encoding as one of the first steps needed.

## Conclusion

From the pilot, it would seem our SNOMED CT encoding method has the potential to become a general purpose terminology encoding approach that can be used in different clinical systems. To achieve this goal, we need to apply this encoding method to a wide range of clinical domains and care settings, so we can continue to refine the methods and tools involved. Most importantly, we need to foster a community of terminology practitioners and researchers who are willing to share their knowledge, tools, contents and experiences in order to accelerate the adoption and use of SNOMED CT across international boundaries.

## Competing interests

The authors declare that they have no competing interests.

## Authors' contributions

All three authors participated in the pilot project. HQ extracted the terms from the Palliative Care Information system while DHL and FYL both were involved in the encoding of the terms. HQ also implemented the SNOMED CT-encoded terms into the Enhanced Palliative Care Information System. DHL and FYL were the primary authors of this paper. All authors have read and approved the final manuscript.

## Pre-publication history

The pre-publication history for this paper can be accessed here:

http://www.biomedcentral.com/1472-6947/10/53/prepub
